# Atypical localization of intraosseous angioleiomyoma in the rib of a pediatric patient: a case report

**DOI:** 10.1186/s12880-018-0297-x

**Published:** 2018-12-19

**Authors:** Goran Djuričić, Zorica Milošević, Tijana Radović, Nataša Milčanović, Predrag Djukić, Marko Radulovic, Jelena Sopta

**Affiliations:** 1 Department of Radiology, University Children’s Hospital, Belgrade, School of Medicine, University of Belgrade, Tiršova 10, 11000 Belgrade, Republic of Serbia; 2 Institute of Oncology and Radiology of Serbia, School of Medicine, University of Belgrade, Pasterova 14, 11000 Belgrade, Republic of Serbia; 3 Institute of Pathology, School of Medicine, University of Belgrade, Dr Subotica 1, 11000 Belgrade, Republic of Serbia

**Keywords:** Intraosseous angioleiomyoma, Rib, Bone tumour, Paediatric

## Abstract

**Background:**

This is the first reported case of a primary intraosseous angioleiomyoma and the second case of a primary leiomyoma of the rib, irrespective of age. Angioleiomyomas mostly occur in patients of advanced age, in any part of the body, particularly the lower extremities and present as painful, slow-growing nodules in the dermis, subcutaneous fat or deep fascia. Other localizations, especially bone, are considered extremely rare, as well as their occurrence in paediatric patients.

**Case presentation:**

A 10-year-old girl was admitted to the orthopaedic surgery department for further assessment of a pain localized in the posterior part of the right hemithorax. After magnetic resonance imaging (MRI) and surgical biopsy, intraosseus angioleiomyoma of the fourth rib was diagnosed by histopathology examination. Atypical costal localization of this type of a benign tumour presents diagnostic difficulty, especially in children. The differential diagnoses included cartilaginous tumours, Ewing sarcoma, fibrous dysplasia, Langerhans cell histiocytosis, intraosseous haemangioma and metastatic tumours. We report a detailed diagnostic procedure including MRI, selective angiography and histopathologic examination.

**Conclusion:**

Diagnosis of intraosseous angioleiomyoma is difficult due to the extreme rarity of this tumour and absence of pathognomonic radiological signs. Although very rarely identified in bones and young age group, radiographers and reporting doctors should be aware of this possible angioleiomyoma presentation and supported by the provided detailed diagnostic information.

## Background

Angioleiomyoma is a rare benign tumour, occurring in the soft tissues (dermis and subcutaneous fat) of the lower extremities in middle-aged women between fourth to sixth decade of life [1] It can arise from the tunica media of a blood vessel in any part of the body and is found in the dermis, subcutaneous fat or deep fascia [1]. The most frequent localization is in the extremities, especially the lower leg (50–70%) [2]. The majority of these lesions appear as solitary, small (< 20 mm), freely moving, subcutaneous, slow-growing nodules, with painful tenderness in approximately 60% of the patients [2, 3]. They occur extremely rarely in bones, where they incite painful swelling [4]. The reported cases of bone localization include the gnathic region, especially the mandible in middle-aged patients [5]. The rarity of intraosseous angioleiomyomas, especially in the pediatric population, renders them very hard to diagnose, occasionally even by histopathological examination.

In our paediatric patient case, primary intraosseus angioleiomyoma was localized in a rib and to our knowledge, this is the first reported case thus far with rib localization and the second case of a primary leiomyoma in the rib. We describe the differential diagnosis of angioleiomyoma at this atypical localization considering the common costal tumour pathologies in children, such as cartilaginous tumours, Ewing sarcoma, fibrous dysplasia, Langerhans cell histiocytosis, intraosseous haemangioma and metastatic tumours.

## Case presentation

A 10-year-old girl was admitted to the orthopaedic surgery department for further assessment of a pain localized in the posterior part of the right hemithorax. According to the physical examination, there was no evidence of a palpable chest wall mass, but the patient reported worsening of symptoms during palpation. Skin and subcutaneous tissue showed no swelling or discoloration. Laboratory values, including serum levels of tumour markers, were all within the normal reference ranges. The initial chest X-ray performed in another institution has been lost. We opted for MRI instead of the CT as the next diagnostic procedure in order to avoid additional exposure of the young patient to ionizing radiation.

**Fig. 1:**
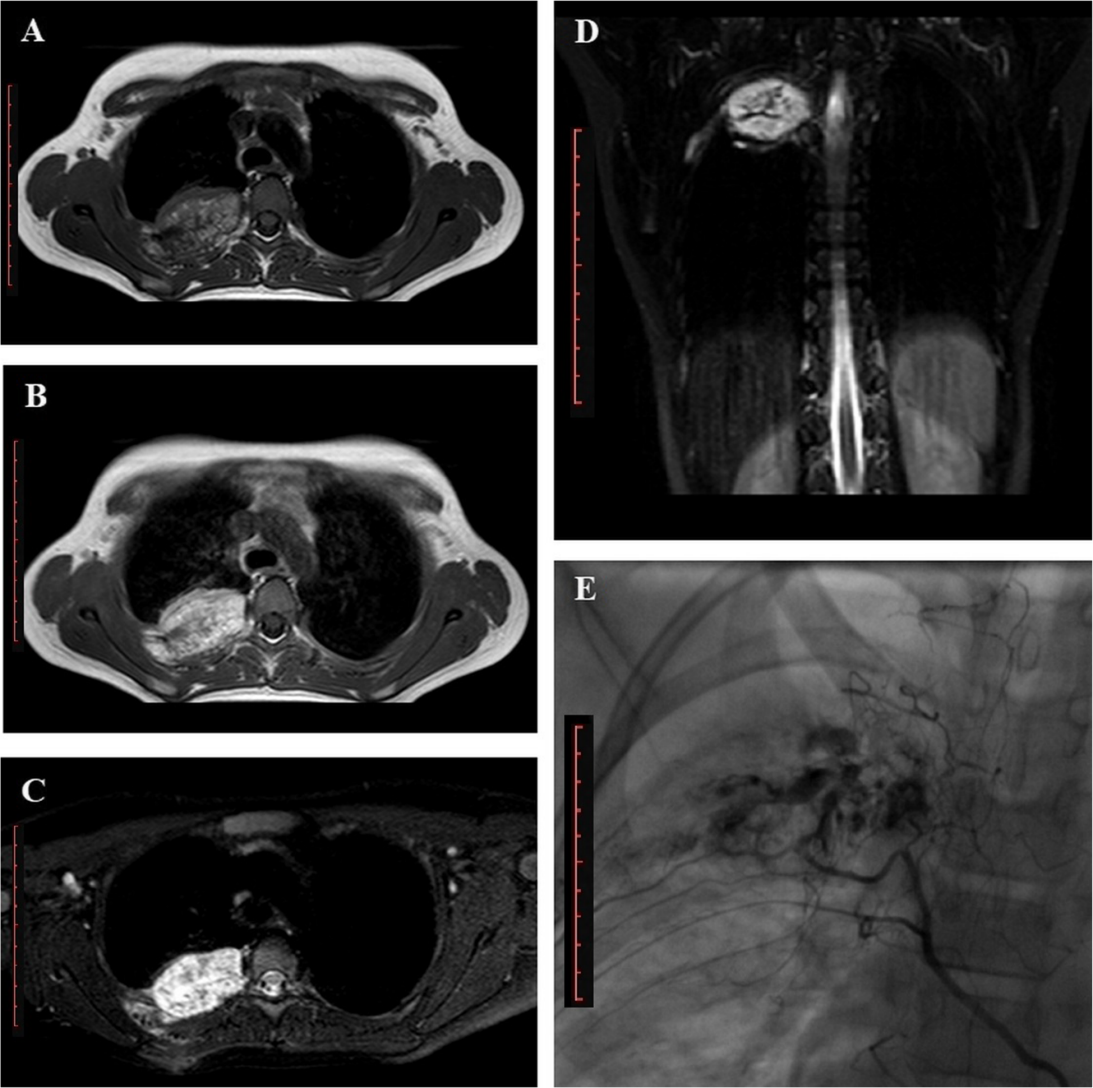
MRI of the thorax reveals a spherical tumour lesion. Rib tumour lesion is presented with lobulated contours, measuring 30 × 50 × 20 mm, located at the medial sector of the posterior arch of the fourth right rib and the adjacent chest wall, at the level of the costovertebral junction. A loss of normal bone structure is notable in the described sector. **a**, Heterogeneous lesion is slightly hyperintense, dominantly isointense to muscle on FSE T1W images; **b**, Intravenous administration of paramagnetic contrast agent resulted in tumour enhancement on T1W images; **c**, On fat-suppressed T2W image a heterogeneous hyperintensity to muscle is obvious; **d**, On fat-suppressed T2W image, two tubular structures, 2.5 mm in size, were seen arising from intercostal blood vessels adjacent to the tumour mass; **e,** Selective angiography revealed a non-homogeneous vascular tumour mass supplied by the three feeding arteries, two of which were proximal and very thin, less than 1 mm in diameter. The diameter of the largest, distal artery was 2.2 mm. *Scale bar*: 10 cm

Magnetic resonance imaging (MRI) of the thorax revealed a spherical, lobulated tumour, located in the posterior arch of the right fourth rib and the adjacent chest wall, 10 mm from its costovertebral junction. The lesion measured 30 × 50 × 20 mm in all three diameters and showed heterogeneous signal intensity. It was mostly hyperintense relative to the muscle on non-contrast T1-weighted (T1W) fast spin echo (FSE) images (Fig. 1a), with prominent postcontrast enhancement on T1-weighted (T1W) fast spin echo (FSE) images (Fig. 1b) and hyperintense on T2W-weighted fat-suppressed (T2W FS) images (Fig. 1c, d). Compression of the adjacent lung parenchyma and thickening of the adjacent pleura was observed as the tumour showed endogenous growth but without signs of lung parenchyma invasion. Vascularization was observed as two vessel branches, 2.5 mm in diameter, arising from the intercostal blood vessels, while the clarity of another feeding branch from the thoracic aorta was limited and only suspected (Fig. 1d). In Fig. 1 (a - d), branching feeding vessels are noted in the center of the lesion. This observation was suggestive of an apparently vascular tumour mass with three feeding arteries but can also be interpreted as distended vascular structure. Those structures are optimally presented in contrast-enhanced T1W in the coronal plane, however, contrast-enhanced T1W was in this case performed only in the axial plane.

**Fig. 2:**
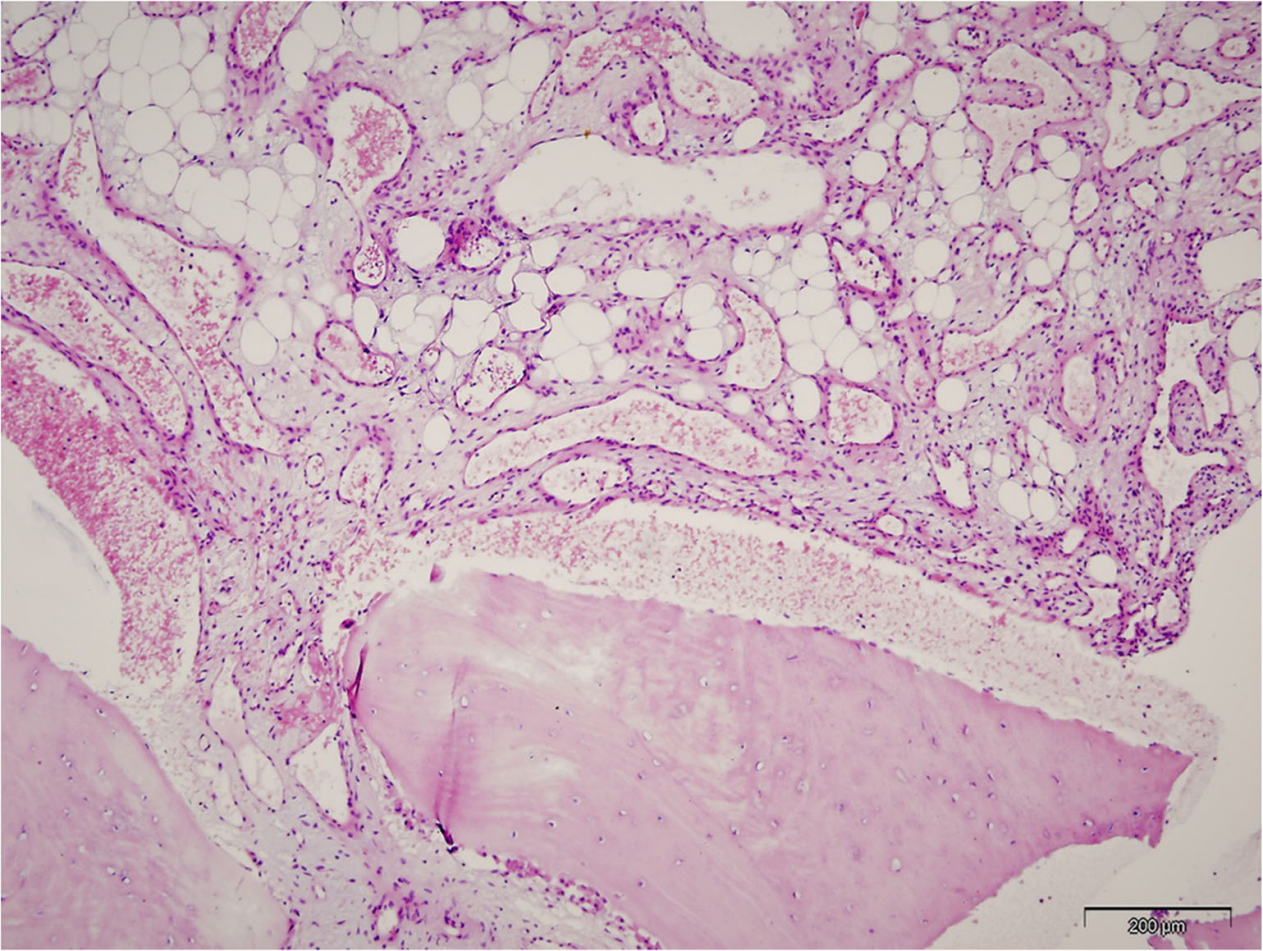
Histological appearance of intraosseous angioleiomyoma, with numerous blood vessels, thick smooth muscle layer and partially irregular lumina. Smooth muscle cells showed no cellular atypia or mitoses. Foci of adipose metaplasia are visible between the blood vessels. *Scale bar*: 200 μm

The patient underwent a surgical biopsy. After an opening of the cortical layer, abnormal bleeding from the rib was seen. Macroscopically, the tumour lesion corresponded to an aneurysmal bone cyst. A specimen was collected, and the material sent for histopathological analysis (Fig. 2). Pathohistological analysis of the tumuor has revealed its numerous blood vessels with a thick wall of smooth muscle layers and partially irregular lumina. Atypical or mitotic smooth muscle cells were not detected. Foci of adipose metaplasia were present in between the blood vessels. The smooth muscle antigen (SMA) immunoreactivity was observed for proliferating perivascular smooth muscle cells and the muscular wall of thick blood vessels, whereas endothelial cells were CD34 positive. These observations were sufficient to establish the diagnosis of intraosseous angioleiomyoma – venous type.

Following the histopathologic diagnosis, selective arteriography was performed in order to plan adequate surgical treatment. The right femoral artery was the route and contrast agent was injected into three catheter tip positions: the ascending aorta, brachiocephalic trunk and descending aorta, in order to visualize all the potential feeding vessels. Non-homogeneous tumour enhancement was obtained only after contrast agent application in the ascending aorta. The three feeding arteries were also visualized – two of them were proximal and very thin, less than 1 mm in diameter, while the distal artery was 2.2 mm in diameter (Fig. 1e). Embolization and surgery were finalized through resection and confirmation on histopathology.

## Discussion and conclusions

This case highlights the extremely unusual angioleiomyoma rib localization and patient age group, with pain without swelling [6]. The absence of a visible or palpable chest mass was yet another unusual trait, possibly due to the endothoracic propagation of the tumour lesion. Intraosseous angioleiomyomas usually arise in the jaw, maxillary tooth socket and the temporal bone [5]. The appendicular skeleton including angioleiomyomas of the tibia, ulna and femoral neck is rarely implicated [7–9]. Therefore, the observed rib localization was striking. Leiomyoma with costal localization was the only reported case of a histologically similar tumour [6].

Chest computed tomography (CT) and MRI are the diagnostic modalities of choice. Whereas CT provides high diagnostic sensitivity for bone and lung parenchyma, MRI is more optimal for assessment of soft tissue propagation. Because of its non-invasiveness, chest MRI has lately become the first step in the diagnostics sequencing of the chest wall tumour assessment for pediatric population. Angioleiomyoma MRI presents as a well-demarcated and strongly enhancing mass with an isointense to slightly hyperintense signal compared to muscle on T1W images and hyperintense signals on T2W images [10]. MRI features of the angioleiomyoma localized in soft tissues of the extremities were described in a series of 8 cases by *Yoo* et al. [11]. According to this study, all the masses were isointense or slightly hyperintense on T1W, whereas they showed slightly higher signal intensity on T2WI relative to skeletal muscle. Moreover, almost all lesions showed homogeneous and good enhancement on postcontrast T1W. Tubular structures adjacent to the masses were found in almost all patients, suggestive of vascular structures, similarly to the case described in the current study [11]. Taken together, all MRI characteristics of the masses were identical to a case described in the current report.

The only case of leiomyoma in the rib was published by *Ganyusufoglu* et al. [6]. They showed MRI characteristics of the mass detected in the 7th left rib with low signal intensity similar to muscle on T1W, whereas the lesion in the current case report showed mostly high signal intensity. In both cases, signal intensity on T2W was prominently high compared to adjacent muscle. Also, both lesions showed homogenous contrast enhancement. However, tubular formations highly suspected of vascular structures were seen only in our case.

Atypical costal localization of angioleiomyoma created a diagnostic challenge, considering that the rib is the common site of several tumours in children, such as cartilaginous tumour, Ewing sarcoma, fibrous dysplasia, Langerhans cell histiocytosis, intraosseous haemangioma and metastatic tumours [12].

Table 1 presents the radiological properties of the described case in comparison to the six plausible diagnoses. Fibrous dysplasia (FD) has been taken into diagnostic consideration as the most frequent benign tumour of the ribs, representing 30% of all benign tumours of the thoracic wall. Up to 20% of the monostotic FD affects ribs, whereby it most frequently affects 2nd rib [13]. That was not the case with our patient whose 4th rib was affected. Good demarcation, heterogeneity and high MRI signal intensity on T2W of the tumour in our patient counted in favor of FD. However, considering that the other typical FD characteristics such as hypo- to isointense lesion on T1W and a sclerotic rim as a hypointense area that surrounds the lesion could not be observed in our patient, FD was excluded as a differential diagnosis [13, 14].

**Table 1:** Radiological properties of the presented case compared to five considered differential diagnoses^a^

	Demarcation	Sclerotic rim	T1W	T2W	T2WFS	Calcification	Postcontrast
Our patient case	Well defined	No	Isointense to slightly hyperintense signal	Hyperintense	Hyperintense	No	Homogenous; Prominently enhanced
Fibrous dysplasia	**Well demarcated**	Yes	Hypointense to isointense	**Hyperintense**	intermediate to high	Yes	Variable contrast enhancement (from mild to marked in degree)
Enchondroma	**Well defined**	**No**	Hypointense	**Hyperintense**	Predominantly high signal	Yes	“Rim and arc” pattern
Ewing sarcoma	Poorly defined	**No**	Hypointense to isointense	Variable signal intensities	Variable signal intensities	**No**	Heterogeneous; Prominently enhanced
Langerhans cell histiocytosis	**Well defined**	**No**	Hypointense to isointense	**Hyperintense**	**Hyperintense**	**No**	Diffusely enhanced
Intraosseous hemangioma	**Well defined**	**No**	Low to intermediate signal intensity (with regions of high signal)	**Hyperintense**	**Hyperintense (with regions of low signal)**	**No**	Heterogeneous Increased enhanced
**Angioleiomyoma**	**Well defined**	**No**	**Isointense to slightly hyperintense** **signal**	**Hyperintense**	**Hyperintense**	**No**	**Homogenous; Prominently enhanced**

^a^Radiological properties matching the presented patient case are marked in bold

Enchondroma was also taken into consideration as a differential diagnosis, having in mind that it is the second most frequent tumour of the ribs [13] and that its MRI characteristics for the most part match the MRI characteristics of the lesion in our patient. Enchondroma is a lesion that consists of a hyaline cartilage lobulus with calcifications within it. It is seen on MRI as a well-defined expansive mass with lobulated contours that is T1W-hypointense and T2W-hyperintense with possible hypointense foci that match calcifications. Due to peripherally localized fibrovascular tissue and presence of avascular hyaline lobes in enchondroma, tumour appearance is contrast-enhanced in a typical “ring and arc” pattern. Since this characteristic of enchondroma was in opposition to a pronounced contrast-enhanced MRI signal intensity of the lesion in our patient, enchondroma was also excluded from differential diagnosis considerations [15–17].

In addition to the most frequent benign rib tumours in children, the most frequent malignant tumours with the same localization in children were also taken into consideration – Ewing sarcoma and metastatic tumours. Ewing sarcoma is the second most frequent malignant bone tumour in children, with a peak incidence in the second decade of life [18, 19]. In general, the extraosseous soft tissue component of Ewing sarcoma tends to be larger compared with its intramedullary component, especially when the flat bones are involved [20]. Heterogenous appearance and hypo- to isointensity on T1W are typical characteristics of Ewing sarcoma that partially matched our case with mostly high signal intensity on T1W. However, unclear demarcation of Ewing sarcoma, “hair on end” appearance of periosteal or reactive bone formation and elevated levels of tumour markers were characteristics that did not match our case. Heterogeneity of a tumour in all sequences could be the result of haemorrhage and tissue necrosis. On fluid sensitive sequences Ewing sarcoma typically shows variable signal intensity, with possible hypointense appearance related to muscles as a consequence of high tumour cellularity, with pronounced contrast-enhanced MRI signal intensity. In our case we observed a notable contrast-enhancement, but with a dominant homogenous contrast-enhanced appearance of the tumour lesion [21, 22].

Langerhans cell histiocytosis matched our case by several MRI characteristics: a demarcated lesion with hyperintense appearance on fluid-sensitive sequences and diffuse contrast enhancement. However, the predominantly hyperintense lesion on T1W in our patient diverged from the typical hypo- to isointense Langerhans cell histiocytosis appearance on T1W [23].

Because of the highly suspected vascular tumour, intraosseous hemangioma was also included in the differential diagnosis. Hemangiomas with bone localization are rare and include less than 1% of all bone tumours [24]. Our case shares similar MRI characteristics with the case published by *Tew* et al. [25]. They reported the case of expansile mass in the right 5th rib proved to be a hemangioma. The lesion was well-defined with a heterogeneous intermediate signal on T1W, a heterogeneous high signal on fat-suppressed T2W and increased contrast enhancement. In contrast to our case, within the lesion there were small areas with signal characteristics of fat [25].

Diagnosis of intraosseous leiomyoma is difficult not only due to the extreme rarity of a tumour but also the absence of pathognomonic radiological signs. The exact diagnosis is thus only achieved by histopathological examination and immunostaining [26]. Differential histopathological diagnosis of angioleiomyoma and other vascular lesions is based on its very consistent case-to-case appearance. Angioleiomyomas are classified into three histological categories: solid (the most common type in soft tissues), venous and cavernous. All three patterns are often observed within the same tumour. These tumours typically exhibit well-defined smooth muscle tissue nodules interspersed with thick-walled blood vessels. The walls of these vessels are composed of the inner and outer layers, while their lumens are partially open. Usually, the smooth muscles of the vessel inner layers are organized in the circumferential pattern. However, the outer layers of the smooth muscles are swirled away from the vessel and integrated with muscle cells at the periphery. It is difficult to determine blood vessels as veins or arteries in these tumours. Although intraosseous localisation is very rare, angioleiomyoma of the bone exhibits similar typical histological features as soft-tissue angioleiomyomas. The use of immunohistochemical staining was helpful for differential diagnosis because the tumour cells were strongly positive for muscle markers, SMA and desmin, thus confirming this tumour’s smooth muscle origin. CD34 was positive in endothelial cells, but negative in the tumour neoplastic cells.

In conclusion, this case emphasizes that atypical intraosseous angioleiomyoma, especially in the ribs of paediatric patients, could mislead the referring physician to more common pathologies in this region. Besides its extreme rarity, diagnosis of intraosseous angioleiomyoma is further complicated by the absence of any pathognomonic radiological signs. Its diagnosis is thus reliably achieved by histopathological examination combined with immunostaining. We expect that the detailed description of this unusual presentation of angioleiomyoma would provide a valuable resource to facilitate diagnosis of such untypical presentations in the future.

## Abbreviations

CE: Contrast enhanced
CT: Computerized tomography
FD: Fibrous dysplasia
FSE: Fast spin echo
LCH: Langerhans cell histiocytosis
MRI: Magnetic resonance imaging
SMA: Smooth muscle antigen
T1W: T1 weighted
T2W: T2 weighted
